# Supplementary material for: Eosinophil Cationic Protein Variation in Patients with Asthma and CRSwNP Treated with Dupilumab
Source: Life (Basel). 2023 Sep 8;13(9):1884. doi: 10.3390/life13091884 (PMC10532820; doi:10.3390/life13091884)
Supplement: Supplementary file 1 [file life-13-01884-s001.zip › life-2532672-supplementary.pdf]

Table S1: Correlation between Serum Eosinophils count and ECP value at each time point. Eos: Eosinophils blood count; ECP: Eosinophils Cationic Protein; RS: Spearman's rank. Data are presented as numbers.

**Time Point**	**EOS (cell/mm³)**	**ECP(U/mL)**	**Correlation**	**RS**
**T0 (Baseline)**	500	69	$p=0.04$	0.42
**T3 months**	860	107	$p<0.0001$	0.96
**T10 months**	300	36.5	$p=0.0004$	0.95
